# Glycemic control and cardiovascular outcomes in patients with diabetes and coronary artery disease according to triglyceride-glucose index: a large-scale cohort study

**DOI:** 10.1186/s12933-023-02112-y

**Published:** 2024-01-06

**Authors:** Zhangyu Lin, Jining He, Sheng Yuan, Chenxi Song, Xiaohui Bian, Min Yang, Kefei Dou

**Affiliations:** 1 Cardiometabolic Medicine Center, Fuwai Hospital, National Center for Cardiovascular Diseases, Chinese Academy of Medical Sciences and Peking Union Medical College, No.167A, Beilishi Road, Xicheng District, Beijing, 100037 China; 2 Department of Cardiology, Fuwai Hospital, National Center for Cardiovascular Diseases, Chinese Academy of Medical Sciences and Peking Union Medical College, No.167A, Beilishi Road, Xicheng District, Beijing, 100037 China; 3 State Key Laboratory of Cardiovascular Disease, Beijing, China; 4 National Clinical Research Center for Cardiovascular Diseases, Beijing, China

**Keywords:** Triglyceride-glucose index, Glycemic control, Diabetes, Coronary artery Disease

## Abstract

**Background:**

The role of triglyceride-glucose (TyG) index, an insulin resistance indicator, in glycemic management for diabetic patients with coronary artery disease (CAD) was still unknown. Therefore, we aimed to explore the association between glycemic control and cardiovascular (CV) outcomes in patients with diabetes and CAD according to different TyG index levels.

**Methods:**

A total of 9996 diabetic patients with angiograph-proven CAD were consecutively recruited from 2017 to 2018 at Fuwai Hospital. Patients were assigned into 3 groups according to TyG index tertiles (T) (T1: <8.895; T2: 8.895-9.400; T3: ≥9.400). According to American Diabetes Association guidelines, controlled glycemia was defined as targeting glycosylated hemoglobin Alc (HbA1c) < 7%. The primary endpoint was CV events including CV death, nonfatal myocardial infarction, and nonfatal stroke.

**Results:**

During a median 3-year follow-up, 381 (3.8%) CV events occurred. Overall, high TyG index (T3) was associated with increased risk of CV events (hazard ratio [HR]: 1.40; 95% confidence interval [CI]: 1.02–1.94) compared with the lowest TyG index (T1) after multivariable adjustment. Upon stratification by the TyG index, in fully adjusted models, controlled glycemia was associated with reduced risk of CV events in the high TyG index (T3) subgroup (HR: 0.64; 95%CI: 0.42–0.96) but not in the low (T1; HR: 0.79; 95%CI: 0.53–1.16) and moderate (T2; HR: 0.84; 95%CI: 0.56–1.25) TyG index subgroups.

**Conclusions:**

Controlled glycemia was associated with improved CV outcomes in patients with diabetes and established CAD, especially in those with high TyG index levels. Our study, for the first time, provided valuable information that TyG index could help making risk stratification on the glycemic management in diabetic patients with CAD.

**Supplementary Information:**

The online version contains supplementary material available at 10.1186/s12933-023-02112-y.

## Introduction

Diabetes is strongly associated with cardiovascular (CV) disease, particularly coronary artery disease (CAD) [1]. Chronic hyperglycemia is closely associated with an increased risk of adverse CV complications, thus reaching low levels of glycemia might improve the clinical outcomes for diabetes patients [2]. However, it was still controversial about glucose control management in diabetes patients with CAD. Several clinical trials indicated that strict glucose control may result in unfavorable clinical outcomes with an increased risk of severe hypoglycemia [1, 3, 4]. Therefore, it is important to identify the specific population that would benefit from glucose control management.

Insulin resistance has been considered as one important determinant of CV risk, which could predict future CV events directly [5]. Triglyceride-glucose (TyG) index, calculated from fasting triglycerides (TG) and fasting blood glucose (FBG) levels, has recently been proposed as a simple and reliable indicator of insulin resistance [6]. TyG index has been identified as a biomarker in predicting the prevalence and prognosis of CAD in the cohorts of CAD primary and secondary prevention population [7–11].

However, the role of TyG in the glycemic control for diabetes patients with CAD remained unclear. Therefore, this study aimed to explore the relationship among TyG index, glycemic control status, and adverse CV events in diabetes patients with angiography-proven CAD.

## Methods

### Study design and population

This was a prospective cohort study conducted at Fuwai Hospital, Chinese Academy of Medical Sciences. The study protocol was complied with the Declaration of Helsinki and approved by the central ethics committee of Fuwai Hospital. Informed consent was obtained from all participants before the study was initiated.

Overall, from January 2017 to December 2018, 13,506 diabetes patients with angiography-proven CAD were consecutively recruited. Diabetes was recorded if the patient had a history of diabetes, received glucose-lowering therapy, had an FBG ≥ 7.0 mmol/L, glycosylated hemoglobin Alc (HbA1c) ≥ 6.5%, or 2 h plasma glucose ≥ 11.1 mmol/L in an oral glucose tolerance test [12]. Angiography-proven CAD was defined as the presence of coronary stenosis ≥ 50% at least one major artery segment assessed by two experienced physicians according to the results of coronary angiography. Major exclusion criteria included missing detailed laboratory data (fasting TG and FBG), age < 18 or ≥ 80 years, severe hepatic or kidney dysfunction, decompensated heart failure, systemic inflammatory disease, malignant tumor, or acute infection.

### Laboratory tests, echocardiography, and definition

On admission, blood samples were obtained from the cubital vein of each participation after at least 12 h of fasting. The concentrations of TG, total cholesterol (TC), high-density lipoprotein cholesterol (HDL-C), FBG, and creatinine were analyzed in an enzymatic assay by automated biochemical analyzer (Hitachi 7150, Tokyo, Japan). Low-density lipoprotein cholesterol (LDL-C) was calculated via the Friedewald method [13]. HbA1c was measured with high-performance liquid chromatography (Tosoh G8 HPLC Analyzer; Tosoh Bioscience, Tokyo, Japan). The hsCRP was examined with standard biochemical techniques at the core laboratory of Fuwai Hospital. The estimated glomerular filtration rate (eGFR) was calculated using the Chinese-modified MDRD (Modification of Diet in Renal Disease) equation [14]. The modified biplane Simpson rule was used to assess left ventricular ejection fraction (LVEF) at rest [15].

The TyG index was calculated according to the following formula: Ln [fasting TG (mg/dL) × FBG (mg/dL)/2] [16], and patients would be categorized according to baseline TyG tertiles (tertile 1 [T1]: <8.895; T2: 8.895-9.400 and T3: ≥9.400). According to the latest American Diabetes Association guideline, controlled glycemia was defined as targeting HbA1c levels less than 7% [17]. Hypertension was defined as systolic blood pressure ≥ 140 mmHg, diastolic blood pressure ≥ 90 mmHg, or the use of antihypertensive therapy [18].

### Evaluation of CAD characteristics and management

Coronary angiogram was performed according to standard techniques by experienced interventional cardiologists. Two independent experienced interventional cardiologists reviewed angiographic data from the catheter laboratory of Fuwai Hospital and recorded the characteristics of CAD, including unique types of coronary stenosis, and the SYNergy between percutaneous coronary intervention with TAXus and cardiac surgery (SYNTAX) score.

### Follow-up and clinical endpoints

Patients were followed up at 6-month intervals until 3-year duration after discharge from medical records, clinical visits, and/or telephone interviews by trained investigators who were blinded to the clinical data. The primary endpoint was CV events (a composite of CV death, nonfatal myocardial infarction [MI], and nonfatal stroke), and the major adverse cardiovascular event (MACE, a composite of CV death and nonfatal MI). The secondary endpoint was CV death, nonfatal MI, and nonfatal stroke. Death was considered CV-caused unless unequivocal non-CV cause could be established. Nonfatal MI was defined as positive cardiac troponins with typical chest pain, typical electrocardiogram serial changes, identification of an intracoronary thrombus by angiography or autopsy, or imaging evidence of new loss of viable myocardium or a new regional wall-motion abnormality [19]. Nonfatal stroke was defined as a new focal neurological deficit lasting > 24 h confirmed by imaging evidence. The endpoints were confirmed by at least two professional physicians.

### Statistical analysis

Continuous variables were expressed as mean ± SD. Categorical variables were presented as number (percentage). The Kolmogorov-Smirnov test was used to test the distribution pattern. The differences of baseline characteristics between groups were analyzed with the Student’s t-test, Mann-Whitney U test, Kruskal-Wallis H test, χ^2^-test, or Fisher exact test where appropriate. The cumulative incidence of clinical endpoints among groups was illustrated by the Kaplan-Meier curves and compared by the log-rank test. Restricted cubic spline (RCS) plots adjusted for age and sex were created to assess linearity assumptions of the relationship between TyG index and clinical endpoints. Univariable and multivariable Cox regression models were used to calculate the hazard ratios (HRs) and 95% confidence intervals (CIs). Potential confounding factors included in the multivariable Cox regression model were age, male sex, BMI, acute coronary syndrome (ACS) presentation, family history of CAD, previous MI, previous revascularization, hypertension, previous stroke, peripheral artery disease, current smoker, LVEF, serum creatinine, TC, HDL-C, LDL-C, hsCRP, SYNTAX score, chronic total occlusion lesion, aspirin use, statins use and insulin use. Subsequently, the relationships between the glycemic control status (controlled or uncontrolled glycemia) and CV events were evaluated according to TyG tertiles to explore the potential effect of TyG index on this association. These above analyses were made for the first subsequent event for all participants. Two tailed *P* values < 0.05 were considered as statistically significant. All statistical analyses were performed using R version 4.0.2 (The R Foundation).

## Results

### Baseline characteristics

**Fig. 1:**
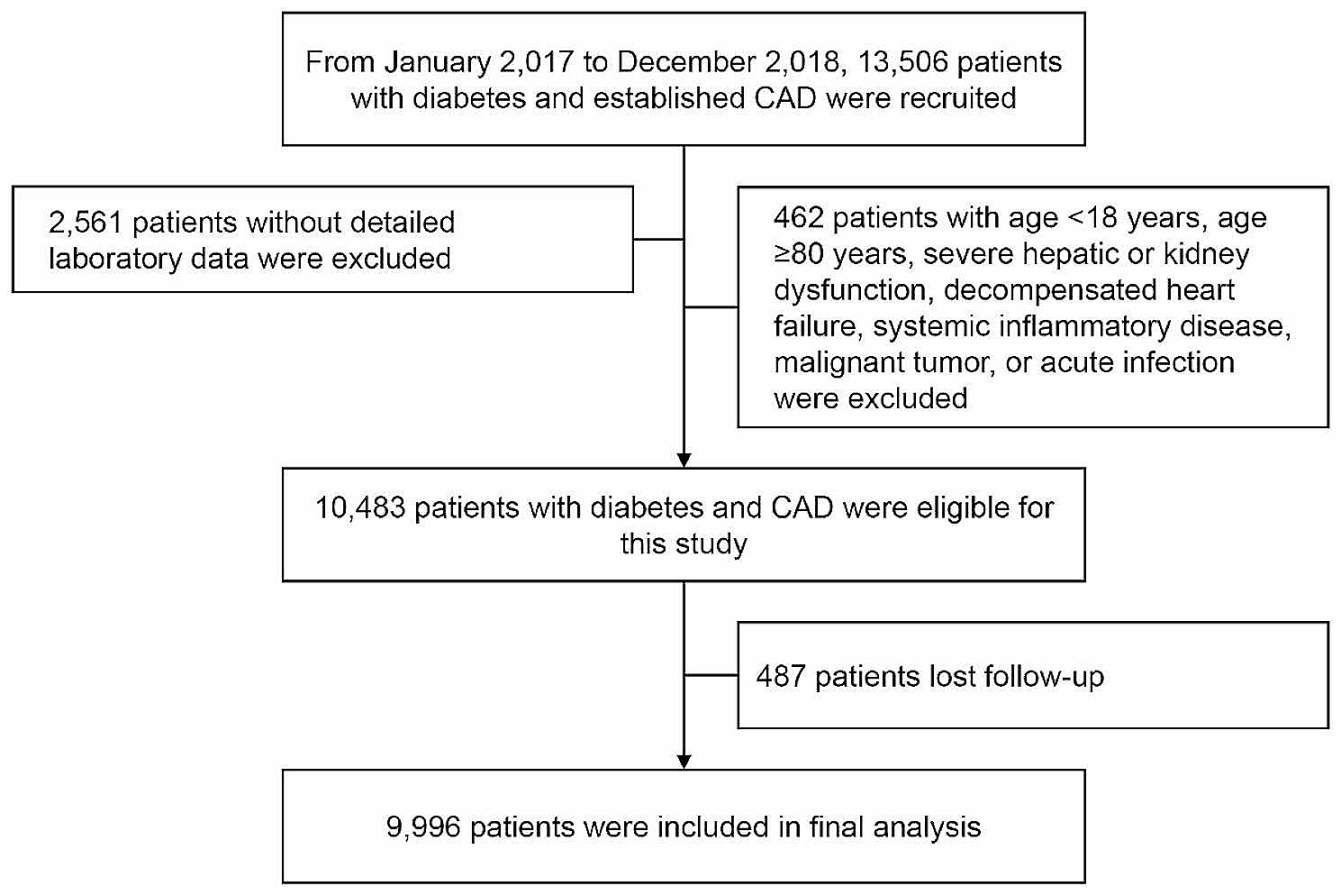
Study flowchart. CAD, oronary artery disease

Finally, a total of 9996 patients were included (Fig. 1). The average age was 60.29 ± 9.27 years, 7502 (75.1%) patients were men, 6945 (69.5%) patients suffered with hypertension, and 3053 (30.5%) patients were current smokers (Table 1). According to the glycemic control status, all patients were divided as uncontrolled glycemia (N = 5583), and controlled glycemia (N = 4413). Overall, patients with uncontrolled glycemia tended to be younger and more current smokers, had higher levels of TyG index, BMI, HbA1c, FBG, TG, TC, LDL-C, hsCRP, SYNTAX score, and were more likely to be involved with three-vessel disease (Table 1). Then, all participants were also separated into 3 groups based on TyG tertiles (T1: N = 3329; T2: N = 3333; T3: N = 3334), whose detailed baseline data were shown in Table 2. The TyG index T3 patients were more likely to be younger females, presented as ACS and current smokers, and had higher levels of serum creatinine, FBG, HbA1c, TC, TG, LDL-C, and hsCRP. The levels of HDL-C were negatively correlated with levels of TyG. Furthermore, continuous TyG index was significantly correlated with traditional CV risk factors, including age, BMI, FBG, HbA1c, TC, TG, LDL-C, HDL-C, hsCRP, and serum creatinine (Table S1).

**Table 1:** Baseline characteristics according to glycemic control status

Characteristics^a^	Overall N = 9996	Uncontrolled glycemia N = 5583	Controlled glycemia N = 4413	*P* value
TyG index	9.19 ± 0.63	9.33 ± 0.64	9.00 ± 0.56	< 0.001
Age, years	60.29 ± 9.27	60.12 ± 9.30	60.51 ± 9.22	0.036
Male	7502 (75.1)	4082 (73.1)	3420 (77.5)	< 0.001
BMI, kg/m^2^	26.31 ± 3.21	26.47 ± 3.26	26.11 ± 3.15	< 0.001
Clinical presentation				0.977
CCS	3790 (37.9)	2118 (37.9)	1672 (37.9)	
ACS	6206 (62.1)	3465 (62.1)	2741 (62.1)	
Family history of CAD	1172 (11.7)	655 (11.7)	517 (11.7)	1.000
Prior MI	2623 (26.2)	1507 (27.0)	1116 (25.3)	0.057
Prior revascularization^b^	3047 (30.5)	1713 (30.7)	1334 (30.2)	0.640
Hypertension	6945 (69.5)	3848 (68.9)	3097 (70.2)	0.183
Prior stroke	1497 (15.0)	849 (15.2)	648 (14.7)	0.484
PAD	743 (7.4)	412 (7.4)	331 (7.5)	0.849
Current smoker	3053 (30.5)	1811 (32.4)	1242 (28.1)	< 0.001
CKD	229 (2.3)	138 (2.5)	91 (2.1)	0.196
LVEF, %	61.57 ± 6.85	61.33 ± 6.97	61.88 ± 6.68	< 0.001
**Laboratory tests**				
Serum creatinine, µmol/L	82.89 ± 18.11	83.03 ± 18.44	82.72 ± 17.70	0.391
eGFR, ml/min/m^2^	85.27 ± 18.65	84.82 ± 19.03	85.83 ± 18.15	0.007
HbA1c, %	7.41 ± 1.29	8.24 ± 1.13	6.36 ± 0.42	< 0.001
FBG, mmol/L	8.12 ± 2.80	9.11 ± 3.09	6.85 ± 1.67	< 0.001
TG, mmol/L	1.83 ± 1.24	1.90 ± 1.34	1.73 ± 1.10	< 0.001
TC, mmol/L	4.00 ± 1.07	4.06 ± 1.09	3.92 ± 1.04	< 0.001
HDL-C, mmol/L	1.07 ± 0.28	1.06 ± 0.28	1.09 ± 0.29	< 0.001
LDL-C, mmol/L	2.38 ± 0.90	2.43 ± 0.91	2.32 ± 0.88	< 0.001
hsCRP, mg/L	2.66 ± 3.03	2.86 ± 3.12	2.41 ± 2.89	< 0.001
**Angiographic data**				
SYNTAX score	12.71 ± 5.47	12.84 ± 5.56	12.55 ± 5.34	0.002
Left main disease	849 (8.5)	493 (8.8)	356 (8.1)	0.186
Three-vessel disease	4765 (47.7)	2784 (49.9)	1981 (44.9)	< 0.001
CTO lesion	1056 (10.6)	590 (10.6)	466 (10.6)	1.000
Thrombotic lesion	202 (2.0)	95 (1.7)	107 (2.4)	0.013
Ostial lesion	1205 (12.1)	697 (12.5)	508 (11.5)	0.146
Type B2/C lesion	7460 (74.6)	4189 (75.0)	3271 (74.1)	0.310
Severe calcification	359 (3.6)	208 (3.7)	151 (3.4)	0.449
**Medications**				
Aspirin	7351 (73.5)	4102 (73.5)	3249 (73.6)	0.884
Statins	9688 (96.9)	5404 (96.8)	4284 (97.1)	0.450
ACEI/ARB	2841 (28.4)	1597 (28.6)	1244 (28.2)	0.664
β-blocker	8981 (89.8)	5071 (90.8)	3910 (88.6)	< 0.001
Diabetic therapy				
Diet control	892 (8.9)	361 (6.5)	531 (12.0)	< 0.001
Oral medication	5081 (50.8)	3193 (57.2)	1888 (42.8)	< 0.001
Insulin use	1756 (17.6)	1397 (25.0)	359 (8.1)	< 0.001

^a^Values are expressed as mean ± standard deviation and count (percentage)

^b^revascularization included percutaneous coronary intervention and coronary artery bypass grafting

*TyG* triglyceride-glucose, *BMI* body mass index, *CCS* chronic coronary syndrome, *ACS* acute coronary syndrome, *MI* myocardial infarction, *CAD* coronary artery disease, *PAD* peripheral artery disease, *LVEF* left ventricular ejection faction, *FBG* fasting blood glucose, *HbA1c* glycosylated hemoglobin A1c, *TC* total cholesterol, *TG* triglyceride, *HDL-C* high-density lipoprotein cholesterol, *LDL-C* low-density lipoprotein cholesterol, *hsCRP* high-sensitivity C-reactive protein, *eGFR* estimated glomerular filtration rate, *SYNTAX* SYNergy between percutaneous coronary intervention with TAXus and cardiac surgery, *CTO* chronic total occlusion, *ACEI* angiotensin converting enzyme inhibitor, *ARB* angiotensin II receptor blocker

**Table 2:** Baseline characteristics according to TyG tertiles

Characteristics^a^	TyG index tertiles			*P* value
	T1 < 8.895 N = 3329	T2 [8.895, 9.400) N = 3333	T3 ≥ 9.400 N = 3334	
TyG index	8.54 ± 0.28	9.14 ± 0.14	9.88 ± 0.42	< 0.001
Age, years	61.66 ± 9.03	60.41 ± 8.99	58.82 ± 9.54	< 0.001
Male	2597 (78.0)	2511 (75.3)	2394 (71.8)	< 0.001
BMI	25.72 ± 3.19	26.44 ± 3.14	26.76 ± 3.23	< 0.001
Clinical presentation				< 0.001
CCS	1345 (40.4)	1263 (37.9)	1182 (35.5)	
ACS	1984 (59.6)	2070 (62.1)	2152 (64.5)	
Family history of CAD	367 (11.0)	403 (12.1)	402 (12.1)	0.306
Prior MI	847 (25.4)	865 (26.0)	911 (27.3)	0.196
Prior revascularization^b^	1044 (31.4)	972 (29.2)	1031 (30.9)	0.119
Hypertension	2261 (67.9)	2337 (70.1)	2347 (70.4)	0.055
Prior stroke	519 (15.6)	500 (15.0)	478 (14.3)	0.358
PAD	304 (9.1)	245 (7.4)	194 (5.8)	< 0.001
Current smoker	901 (27.1)	1018 (30.5)	1134 (34.0)	< 0.001
CKD	49 (1.5)	74 (2.2)	106 (3.2)	< 0.001
LVEF, %	61.78 (6.79)	61.47 (6.81)	61.47 (6.95)	0.108
**Laboratory tests**				
Serum creatinine, µmol/L	81.68 ± 16.75	82.67 ± 17.77	84.33 ± 19.60	< 0.001
eGFR, ml/min/m^2^	86.45 ± 17.04	85.42 ± 18.60	83.93 ± 20.11	< 0.001
HbA1c, %	6.99 ± 1.05	7.32 ± 1.17	7.91 ± 1.45	< 0.001
FBG, mmol/L	6.53 ± 1.41	7.79 ± 1.90	10.03 ± 3.40	< 0.001
TG, mmol/L	1.04 ± 0.28	1.59 ± 0.39	2.85 ± 1.64	< 0.001
TC, mmol/L	3.57 ± 0.86	3.96 ± 0.98	4.46 ± 1.16	< 0.001
HDL-C, mmol/L	1.15 ± 0.31	1.07 ± 0.27	1.00 ± 0.24	< 0.001
LDL-C, mmol/L	2.11 ± 0.74	2.43 ± 0.87	2.61 ± 1.00	< 0.001
hsCRP, mg/L	2.35 ± 2.90	2.66 ± 3.01	2.98 ± 3.14	< 0.001
**Angiographic data**				
SYNTAX score	12.54 ± 5.23	12.88 ± 5.64	12.71 ± 5.53	0.035
Left main disease	301 (9.0)	290 (8.7)	258 (7.7)	0.141
Three-vessel disease	1583 (47.6)	1593 (47.8)	1589 (47.7)	0.98
CTO lesion	313 (9.4)	378 (11.3)	365 (10.9)	0.025
Thrombotic lesion	45 (1.4)	65 (2.0)	92 (2.8)	< 0.001
Ostial lesion	400 (12.0)	418 (12.5)	387 (11.6)	0.502
Type B2/C lesion	2464 (74.0)	2493 (74.8)	2503 (75.1)	0.588
Severe calcification	143 (4.3)	107 (3.2)	109 (3.3)	0.028
**Medications**				
Aspirin	2505 (75.2)	2501 (75.0)	2345 (70.3)	< 0.001
Statins	3215 (96.6)	3234 (97.0)	3239 (97.2)	0.359
ACEI/ARB	919 (27.6)	968 (29.0)	954 (28.6)	0.41
β-blocker	2913 (87.5)	2974 (89.2)	3094 (92.8)	< 0.001
Diabetic therapy				
Diet control	337 (10.1)	304 (9.1)	251 (7.5)	0.001
Oral medication	1741 (52.3)	1626 (48.8)	1714 (51.4)	0.012
Insulin use	526 (15.8)	541 (16.2)	689 (20.7)	< 0.001

^a^Values are expressed as mean ± standard deviation and count (percentage)

^b^Revascularization included percutaneous coronary intervention and coronary artery bypass grafting

Abbreviations as in Table 1

### TyG and adverse CV events risk

During a median follow-up of 3.1 (IQR: 3.0-3.3) years, a total of 381 CV events and 328 MACEs were recorded (Table 3). Compared with non-event participations, patients suffered with CV events were more likely to be older, suffering with hypertension, presented as ACS, and had higher levels of TyG index, FBG, HbA1c, serum creatinine, and hsCRP (Table S2).

**Table 3:** Cox regression analysis of TyG index with clinical endpoints

TyG tertiles	Events (%)	Univariable analysis		Multivariable analysis^c^	
		HR (95%CI)	*P* value	HR (95%CI)	*P* value
**CV events** ^**a**^	381(3.8)	1.27 (1.09–1.48)	0.002	1.78 (1.35–2.35)	< 0.001
T1	112 (3.4)	Reference	-	Reference	-
T2	121 (3.6)	1.07 (0.83–1.39)	0.585	1.10 (0.84–1.45)	0.474
T3	148 (4.4)	1.32 (1.03–1.69)	0.026	1.40 (1.02–1.94)	0.040
**MACEs** ^**b**^	328 (3.3)	1.29 (1.09–1.52)	0.003	1.93 (1.43–2.60)	< 0.001
T1	94 (2.8)	Reference	-	Reference	-
T2	104 (3.1)	1.10 (0.83–1.45)	0.504	1.15 (0.85–1.54)	0.363
T3	130 (3.9)	1.38 (1.06–1.80)	0.017	1.55 (1.09–2.20)	0.016

^a^CV events were defined as a composite of CV death, nonfatal MI and nonfatal stroke

^b^MACEs were defined as a composite of CV death and nonfatal MI

^c^Models adjusted for age, male sex, BMI, ACS presentation, family history of CAD, previous MI, previous revascularization, hypertension, previous stroke, PAD, current smoker, LVEF, serum creatinine, TC, HDL-C, LDL-C, hsCRP, SYNTAX score, CTO lesion, aspirin use, statins use and insulin use

*HR* hazard ratio, *CI* confidence interval, *CV* cardiovascular, *MACE* major adverse cardiovascular event, other abbreviations as in Table 1

**Fig. 2:**
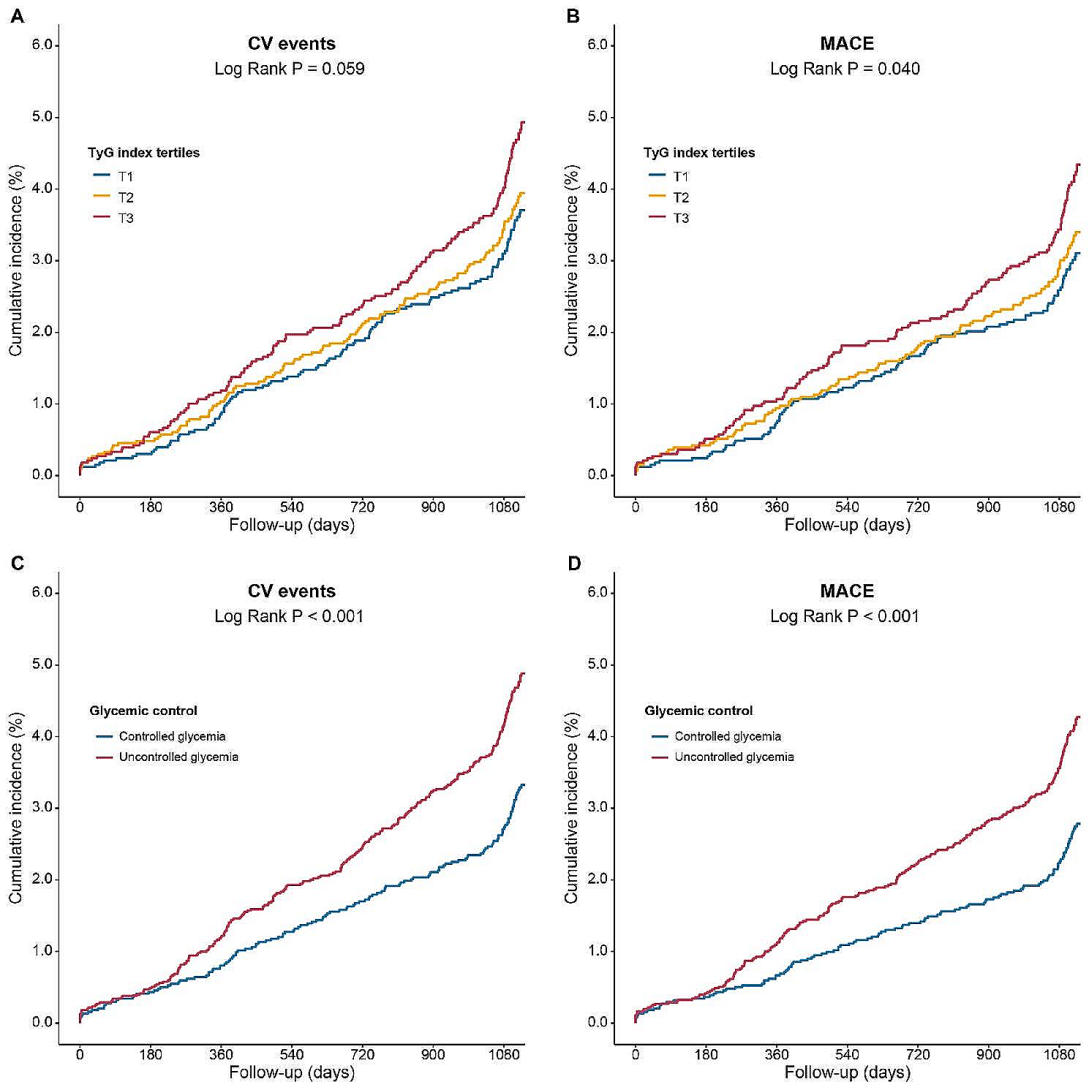
Kaplan-Meier curves of TyG index tertiles for (**A**) CV events, (**B**) MACEs and glycemic control status for (**C**) CV events, (**D**) MACEs. Abbreviations as in Tables 1 and 3

The prevalence of CV events in the TyG T1, T2, and T3 groups were 112 (3.4%), 121 (3.6%), and 148 (4.4%) respectively, and the prevalence of MACEs in the TyG T1, T2, and T3 groups were 94 (2.8%), 104 (3.1%), and 130 (3.9%) respectively (Table 3). Kaplan-Meier survival analyses showed a significant difference in the incidence of CV events and MACEs among the 3 groups at the 3-year follow-up, with the highest CV events and MACEs rate in TyG T3 (all *P* values < 0.001, Fig. 2).

Then, RCS analyses indicated that there were positive linear associations of the TyG with the CV events and MACE rate at 3-year follow-ups even after adjustment for age and sex (all *P* values for nonlinearity > 0.05) (Figure S1). The multivariable cox regression analyses results showed that in comparisons with TyG T1 subjects, the multivariable-adjusted HR for CV events and MACEs at the 3-year follow-up were 1.40 (95% CI: 1.02–1.94) and 1.55 (95% CI: 1.09–2.20) for TyG T3 subjects respectively. No difference could be seen for the risk of CV events or MACEs between TyG T1 and T2 subjects. Moreover, the HR per unit increase of TyG in predicted CV event was 1.78 (95%CI: 1.35–2.35), and in predicted MACE was 1.93 (1.43–2.60) (Table 3). Relationship between TyG tertiles and secondary endpoints could be obtained in Table S3.

### Glycemic control and adverse CV events according to different TyG tertiles

**Fig. 3:**
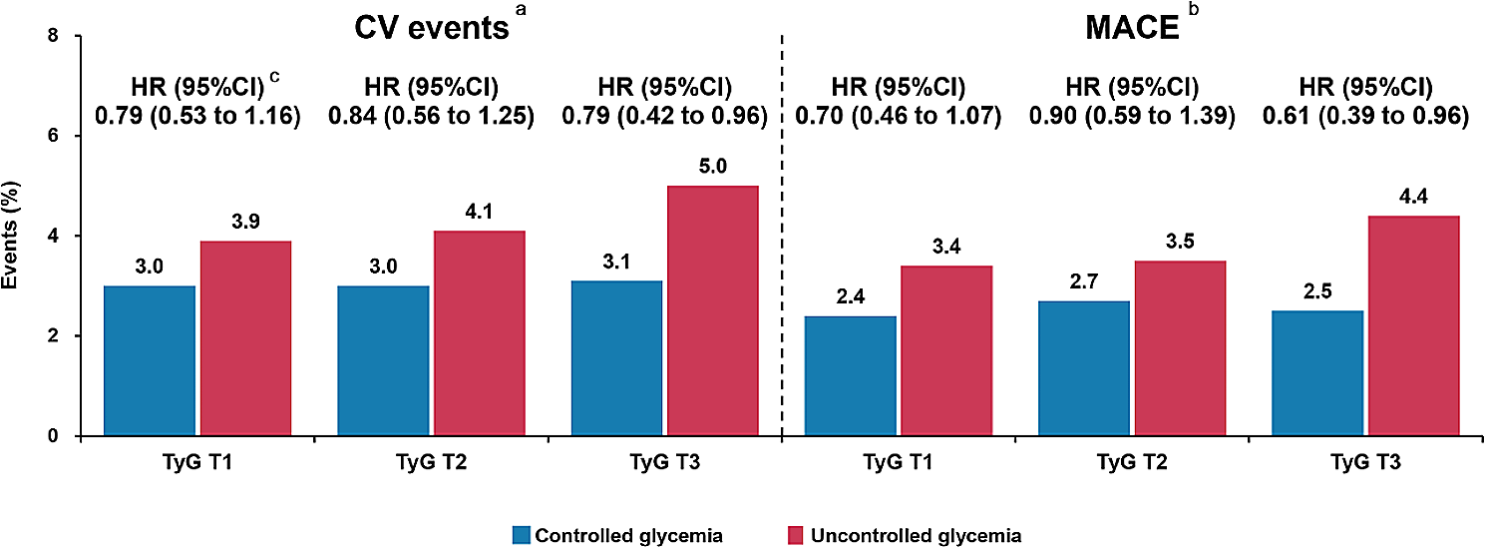
Incidence of study endpoints. ^a^CV events were defined as a composite of CV death, nonfatal MI and nonfatal stroke. ^b^MACE was defined as a composite of CV death and nonfatal MI. ^c^HR with 95%CI was estimated by multivariable Cox regression models adjusted for age, male sex, BMI, ACS presentation, family history of CAD, previous MI, previous revascularization, hypertension, previous stroke, PAD, current smoker, LVEF, serum creatinine, TC, HDL-C, LDL-C, hsCRP, SYNTAX score, CTO lesion, aspirin use, statins use and insulin use. Abbreviations as in Tables 1 and 3

Overall, patients with controlled glycemia had lower prevalence of CV events, MACEs and non-fatal MI than those with uncontrolled glycemia (all *P* < 0.05) (Table 4). Table 5 and Fig. 3 shown the results of stratification multivariable Cox regression analysis of glycemic control status and CV events according to different TyG tertiles. For TyG T3 patients, controlled glycemia were significantly associated with lower risk of CV events (HR, 0.64; 95%CI: 0.42–0.96) and MACEs (HR, 0.61; 95%CI, 0.39–0.96) than those with uncontrolled glycemia. While, for those with TyG T1 or T2, no significantly difference of risk of CV events and MACEs was observed between controlled or uncontrolled glycemia groups (all *P* > 0.05). Glycemic control in relation to the secondary endpoints according to TyG tertiles could be obtained in Table S4.

**Table 4:** Cox regression analysis of glycemic control status with clinical endpoints

Endpoints	Events (%)		Univariable analysis		Multivariable analysis^c^	
	Controlled	Uncontrolled	HR (95%CI)	*P* value	HR (95%CI)	*P* value
**CV events** ^**a**^	133 (3.0)	248 (4.4)	0.67 (0.55–0.83)	< 0.001	0.70 (0.56–0.87)	0.001
**MACEs** ^**b**^	111 (2.5)	217 (3.9)	0.64 (0.51–0.81)	< 0.001	0.67 (0.53–0.85)	< 0.001
**CV death**	57 (1.3)	100 (1.8)	0.72 (0.52-1.00)	0.047	0.72 (0.51–1.01)	0.057
**Nonfatal MI**	40 (0.9)	93 (1.7)	0.54 (0.37–0.78)	0.001	0.59 (0.41–0.87)	0.007
**Nonfatal stroke**	22 (0.5)	32 (0.6)	0.87 (0.50–1.49)	0.608	0.85 (0.49–1.50)	0.583

^a^CV events were defined as a composite of CV death, nonfatal MI and nonfatal stroke

^b^MACEs were defined as a composite of CV death and nonfatal MI

^c^Models adjusted for age, male sex, BMI, ACS presentation, family history of CAD, previous MI, previous revascularization, hypertension, previous stroke, PAD, current smoker, LVEF, serum creatinine, TC, HDL-C, LDL-C, hsCRP, SYNTAX score, CTO lesion, aspirin use, statins use and insulin use

Abbreviations as in Tables 1 and 3

**Table 5:** Glycemic control in relation to study endpoints according to TyG index tertiles

TyG tertiles	Glycemic control Events (%)		Univariable analysis		Multivariable analysis^c^	
	Controlled	Uncontrolled	HR (95%CI)	*P* value	HR (95%CI)	*P* value
**CV events** ^**a**^						
TyG T1	59 (3.0)	53 (3.9)	0.77 (0.53–1.12)	0.167	0.79 (0.53–1.16)	0.224
TyG T2	45 (3.0)	76 (4.1)	0.72 (0.50–1.04)	0.083	0.84 (0.56–1.25)	0.382
TyG T3	29 (3.1)	119 (5.0)	0.61 (0.40–0.91)	0.016	0.64 (0.42–0.96)	0.033
**MACEs** ^**b**^						
TyG T1	47 (2.4)	47 (3.4)	0.69 (0.46–1.03)	0.072	0.70 (0.46–1.07)	0.101
TyG T2	40 (2.7)	64 (3.5)	0.76 (0.51–1.13)	0.182	0.90 (0.59–1.39)	0.639
TyG T3	24 (2.5)	106 (4.4)	0.56 (0.36–0.88)	0.011	0.61 (0.39–0.96)	0.034

^a^CV events were defined as a composite of CV death, nonfatal MI and nonfatal stroke

^b^MACEs were defined as a composite of CV death and nonfatal MI

^c^Models adjusted for age, male sex, BMI, ACS presentation, family history of CAD, previous MI, previous revascularization, hypertension, previous stroke, PAD, current smoker, LVEF, serum creatinine, TC, HDL-C, LDL-C, hsCRP, SYNTAX score, CTO lesion, aspirin use, statins use and insulin use

Abbreviations as in Tables 1 and 3

## Discussion

In this study, the association among TyG index, glycemic control, and adverse CV events in diabetes patients with angiography-proven CAD was evaluated, revealing that uncontrolled glycemia was significantly associated with CV events and MACEs in high TyG (T3) patients, while those association could not be seen in patients with low TyG index (T1 or T2). Our study demonstrated, for the first time, that the association between glycemic control and adverse CV events was more pronounced in high TyG patients, suggesting TyG could help making risk stratification when considering glycemic control for diabetes patients combined with CAD.

It still remained controversies about the impact of glycemic control on CV events. Several cardiovascular outcome trials, such as VADT [20], ACCORD [21], and ADVANCE [22], failed to find a significant reduction of CV events risk when comparing more strict glycemic control with the standard care of diabetes. However, certain studies, such as the DCCT/EDIC [23] and UKPDS [24] study, have demonstrated that strict glycemic control might reduce the incidence of CV events. Identifying patients who are more likely to benefit from glycemic control management might help to resolve this problem.

Insulin secretion and resistance play important roles in glycemic control and might further influence the CV outcomes of diabetic patients [25]. The higher degree of insulin resistance might induce insufficient insulin secretion after treatment with glycemic control agents, especially those targeting in improving insulin secretion, and result in a poor response in glucose control management, which might lead to persistent hyperinsulinemia and hyperglycemia [26]. Hyperinsulinemia might continuously active the growth factor receptor-bound protein-2 signal pathway inactive the insulin receptor substrate pathway, and increase the level of plasminogen activator inhibitor-1, vascular cell adhesion molecule 1, and endothelin-1, which might induce the vasoconstriction, proliferation, migration of endothelium, promote atherosclerotic plaque formation and instability and increase the risk the adverse CV events [27]. On the population level, however, few studies reported the impact of insulin resistance on the association between glycemic control and CV events. To our knowledge, only one small-sample study achieving glycemic control and improving insulin resistance might slightly but not significantly reduce the incidence of CV events for early type 2 diabetes patients [28]. Suitable insulin resistance indices and larger population with higher CV risk might help clarify this question.

The TyG index has been extensively validated as a dependable indicator for evaluating insulin resistance, exhibiting notable sensitivity and specificity. Consequently, it has found widespread application in clinical settings due to its practicality, affordability, and versatile utility [29]. The relationship between TyG index and CAD has been thoroughly examined, and previous cohort studies and meta-analyses have extensively demonstrated its predictive value for a high incidence of CAD [30, 31]. For CAD patients, multiple cohort studies have indicated that individuals with a high TyG index are independently associated with an increased risk of repeated revascularization and in-hospital mortality [32, 33]. For diabetic patients, one previous study has indicated TyG index was associated with the all-cause mortality risk in patients with diabetes or pre-diabetes [34]. However, when it comes to the glycemic control for diabetic patients combined with established CAD, there was no research elucidating the role of the TyG with adverse CV events in this population. Thus, we focused on this point for the first time and revealed that uncontrolled glycemia was associated with an increase in CV risk only in those with high TyG patients.

This study has several limitations. Firstly, this is a single-center observational study. Therefore, it was not feasible to establish a definitive causal relationship between the TyG in conjunction with glycemia control status and the incidence of CV events. Secondly, dynamic changes in the TyG and glycemia control status during follow-up were not presented in our study. It was still unknown about the association between changes of TyG and glycemia control status and prognosis for diabetic CAD population. Thirdly, despite controlling for potential confounders as covariates in multivariable regression models, it is important to acknowledge that the impact of uncollected confounders cannot be completely disregarded. Fourthly, since there were no new hypoglycemic agents (such as glucagon-like peptide-1 receptor agonist, odium-dependent glucose transporters 2 inhibitors) data available, we could not estimate the effect of those new hypoglycemic agents on glycemic control management in this study. Fifthly, the post-hoc analysis of the NID-2 trial demonstrated that the number of main CV risk factors well controlled by drug therapy significantly influences the clinical outcome for diabetic patients with very high CV risk [35]. Although we had tried our best to adjust CV risk factors (including hypertension, TC, HDL-C, LDL-C, aspirin use, statins use, and insulin use) in our study, follow-up data regarding medications (such as antihypertensive agents, lipid‑modulating agents, anticoagulants and anti-diabetic drugs) were not available in this study, which possibly had impacts on CV outcomes. Sixthly, our study only retrospectively analyzed the results of glycemic control and was unable to explore the specific process of glycemic management and the specific changes in HbA1c levels during glycemic management. Detailed glycemic control strategy and HbA1C target assisted by TyG index need to be further confirmed in larger prospective studies. Seventh, although we adjusted all available baseline data of insulin and lipid-lowering drugs, levels of TyG index might be affected by the above medication. Further studies might be needed to confirm the association of the TyG index with lipid-lowering agents and insulin therapy in CAD patients with diabetes. Finally, it was unclear whether glycemic control could improve clinical outcomes by improving insulin resistance. Future prospective and longitudinal studies are needed to explore the impact and mechanism of controlling blood glucose and improving insulin resistance on the clinical outcomes of diabetes patients with established CAD.

## Conclusion

In this study, we firstly found the association between glycemic control status and adverse CV events was more pronounced in high TyG patients, suggesting TyG index could help making risk stratification on glycemic control for diabetes patients combined with CAD.

### Electronic supplementary material

Below is the link to the electronic supplementary material.

**Supplementary Material 1: Table S1.** Correlation analysis between TyG index and clinical risk factors. **Table S2.** Baseline characteristics according to CV events. **Table S3.** TyG tertiles in relation to secondary endpoints. **Table S4.** Glycemic control status in relation to study endpoints according to TyG index tertiles. **Figure S1.** Restrict cubic spine analysis for the association of TyG index with the risk of (A) CV events and (B) MACEs

## Data Availability

Data underlying this article will be shared upon reasonable request and in accordance with the appropriate general data protection regulation (GDPR).

## Abbreviations

ACS: Acute coronary syndrome
ACEI: Angiotensin converting enzyme inhibitor
ARB: Angiotensin II receptor blocker
BMI: Body mass index
CAD: Coronary artery disease
CCS: Chronic coronary syndrome
CI: Confidence interval
CV: Cardiovascular
CVOTs: Cardiovascular outcome trials
CTO: Chronic total occlusion
eGFR: Estimated glomerular filtration rate
FBG: Fasting blood glucose
HDL-C: High-density lipoprotein cholesterol
HbAlc: Glycosylated hemoglobin Alc
HR: Hazard ratio
hsCRP: High-sensitivity C-reactive protein
IDI: integrated discrimination improvement
LVEF: Left ventricular ejection faction
LDL-C: Low-density lipoprotein cholesterol
MI: Myocardial infarction
NRI: Net reclassification improvement
PAD: Peripheral artery disease
RCS: Restricted cubic splines
SYNTAX: SYNergy between percutaneous coronary intervention with TAXus and cardiac surgery
TyG: Triglyceride-glucose
TC: Total cholesterol
TG: Triglycerides

## References

[1] Rodriguez-Gutierrez R, Gonzalez-Gonzalez JG, Zuniga-Hernandez JA, McCoy RG (2019). Benefits and harms of intensive glycemic control in patients with type 2 Diabetes. BMJ.

[2] Selvin E, Marinopoulos S, Berkenblit G, Rami T, Brancati FL, Powe NR, Golden SH (2004). Meta-analysis: glycosylated hemoglobin and Cardiovascular Disease in Diabetes Mellitus. Ann Intern Med.

[3] Miller ME, Williamson JD, Gerstein HC, Byington RP, Cushman WC, Ginsberg HN (2014). Effects of randomization to intensive glucose control on adverse events, Cardiovascular Disease, and mortality in older versus younger adults in the ACCORD Trial. Diabetes Care.

[4] Vijan S, Sussman JB, Yudkin JS, Hayward RA (2014). Effect of patients’ risks and preferences on health gains with plasma glucose level lowering in type 2 Diabetes Mellitus. JAMA Intern Med.

[5] Bonora E, Formentini G, Calcaterra F, Lombardi S, Marini F, Zenari L (2002). HOMA-estimated insulin resistance is an Independent predictor of Cardiovascular Disease in type 2 diabetic subjects: prospective data from the Verona Diabetes Complications Study. Diabetes Care.

[6] Hoshino T, Mizuno T, Ishizuka K, Takahashi S, Arai S, Toi S, Kitagawa K (2022). Triglyceride-glucose index as a prognostic marker after ischemic Stroke or transient ischemic Attack: a prospective observational study. Cardiovasc Diabetol.

[7] Zhu Y, Liu K, Chen M, Liu Y, Gao A, Hu C (2021). Triglyceride-glucose index is associated with in-stent restenosis in patients with acute coronary syndrome after percutaneous coronary intervention with drug-eluting stents. Cardiovasc Diabetol.

[8] Zhang Y, Ding X, Hua B, Liu Q, Gao H, Chen H (2022). High triglyceride-glucose index is Associated with Poor Cardiovascular outcomes in nondiabetic patients with ACS with LDL-C below 1.8 mmol/L. J Atheroscler Thromb.

[9] He J, Bian X, Song C, Zhang R, Yuan S, Yin D, Dou K (2022). High neutrophil to lymphocyte ratio with type 2 Diabetes Mellitus predicts poor prognosis in patients undergoing percutaneous coronary intervention: a large-scale cohort study. Cardiovasc Diabetol.

[10] He J, Yuan S, Song C, Song Y, Bian X, Gao G, Dou K (2023). High triglyceride-glucose index predicts cardiovascular events in patients with coronary bifurcation lesions: a large-scale cohort study. Cardiovasc Diabetol.

[11] He J, Song C, Wang H, Zhang R, Yuan S, Dou K (2023). Diabetes Mellitus with mild or moderate kidney dysfunction is associated with poor prognosis in patients with coronary artery Disease: a large-scale cohort study. Diabetes Res Clin Pract.

[12] ElSayed NA, Aleppo G, Aroda VR, Bannuru RR, Brown FM, Bruemmer D (2023). 2. Classification and diagnosis of Diabetes: standards of Care in Diabetes-2023. Diabetes Care.

[13] Friedewald WT, Levy RI, Fredrickson DS (1972). Estimation of the concentration of low-density lipoprotein cholesterol in plasma, without use of the preparative ultracentrifuge. Clin Chem.

[14] Ma YC, Zuo L, Chen JH, Luo Q, Yu XQ, Li Y (2006). Modified glomerular filtration rate estimating equation for Chinese patients with chronic Kidney Disease. J Am Soc Nephrol.

[15] Schiller NB, Shah PM, Crawford M, DeMaria A, Devereux R, Feigenbaum H (1989). Recommendations for quantitation of the left ventricle by two-dimensional echocardiography. American Society of Echocardiography Committee on standards, Subcommittee on quantitation of two-Dimensional echocardiograms. J Am Soc Echocardiogr.

[16] Guerrero-Romero F, Simental-Mendia LE, Gonzalez-Ortiz M, Martinez-Abundis E, Ramos-Zavala MG, Hernandez-Gonzalez SO (2010). The product of triglycerides and glucose, a simple measure of insulin sensitivity. Comparison with the euglycemic-hyperinsulinemic clamp. J Clin Endocrinol Metab.

[17] ElSayed NA, Aleppo G, Aroda VR, Bannuru RR, Brown FM, Bruemmer D (2023). 6. Glycemic targets: standards of Care in Diabetes-2023. Diabetes Care.

[18] Williams B, Mancia G, Spiering W, Agabiti Rosei E, Azizi M, Burnier M (2018). 2018 ESC/ESH guidelines for the management of arterial Hypertension. Eur Heart J.

[19] Thygesen K, Alpert JS, Jaffe AS, Simoons ML, Chaitman BR, White HD (2012). Third universal definition of Myocardial Infarction. Eur Heart J.

[20] Duckworth W, Abraira C, Moritz T, Reda D, Emanuele N, Reaven PD (2009). Glucose control and vascular Complications in veterans with type 2 Diabetes. N Engl J Med.

[21] Study G, Gerstein HC, Miller ME, Byington RP, Goff DC, Bigger JT, Action to Control Cardiovascular Risk in Diabetes (2008). Effects of intensive glucose lowering in type 2 Diabetes. N Engl J Med.

[22] Group AC, Patel A, MacMahon S, Chalmers J, Neal B, Billot L (2008). Intensive blood glucose control and vascular outcomes in patients with type 2 Diabetes. N Engl J Med.

[23] Diabetes C, Complications Trial /Epidemiology of Diabetes I, Complications Study Research (2016). Intensive Diabetes Treatment and Cardiovascular outcomes in Type 1 Diabetes: the DCCT/EDIC Study 30-Year follow-up. Diabetes Care.

[24] Effect of intensive (1998). Blood-glucose control with metformin on Complications in overweight patients with type 2 Diabetes (UKPDS 34). UK prospective Diabetes study (UKPDS) Group. Lancet.

[25] DeFronzo RA, Ferrannini E (1991). Insulin resistance. A multifaceted syndrome responsible for NIDDM, obesity, Hypertension, dyslipidemia, and atherosclerotic Cardiovascular Disease. Diabetes Care.

[26] Koufakis T, Papanas N, Zebekakis P, Kotsa K (2021). Treatment options following metformin in primary prevention populations with type 2 Diabetes: which is the right road to take?. Expert Rev Clin Pharmacol.

[27] Lambie M, Bonomini M, Davies SJ, Accili D, Arduini A, Zammit V (2021). Insulin resistance in Cardiovascular Disease, uremia, and peritoneal dialysis. Trends Endocrinol Metab.

[28] Li J, Feng Z, Li Q, He Y, Zhao C, He J (2014). Insulin glargine effectively achieves glycemic control and improves insulin resistance in patients with early type 2 Diabetes that exhibit a high risk for Cardiovascular Disease. Exp Ther Med.

[29] Alizargar J, Bai CH, Hsieh NC, Wu SV (2020). Use of the triglyceride-glucose index (TyG) in Cardiovascular Disease patients. Cardiovasc Diabetol.

[30] Tao LC, Xu JN, Wang TT, Hua F, Li JJ (2022). Triglyceride-glucose index as a marker in Cardiovascular Diseases: landscape and limitations. Cardiovasc Diabetol.

[31] Liang S, Wang C, Zhang J, Liu Z, Bai Y, Chen Z (2023). Triglyceride-glucose index and coronary artery Disease: a systematic review and meta-analysis of risk, severity, and prognosis. Cardiovasc Diabetol.

[32] Guo X, Shen R, Yan S, Su Y, Ma L (2023). Triglyceride-glucose index for predicting repeat revascularization and in-stent restenosis in patients with chronic coronary syndrome undergoing percutaneous coronary intervention. Cardiovasc Diabetol.

[33] Zhang R, Shi S, Chen W, Wang Y, Lin X, Zhao Y (2023). Independent effects of the triglyceride-glucose index on all-cause mortality in critically ill patients with coronary Heart Disease: analysis of the MIMIC-III database. Cardiovasc Diabetol.

[34] Zhang Q, Xiao S, Jiao X, Shen Y (2023). The triglyceride-glucose index is a predictor for cardiovascular and all-cause mortality in CVD patients with Diabetes or pre-diabetes: evidence from NHANES 2001–2018. Cardiovasc Diabetol.

[35] Sasso FC, Simeon V, Galiero R, Caturano A, De Nicola L, Chiodini P (2022). The number of risk factors not at target is associated with cardiovascular risk in a type 2 diabetic population with albuminuria in primary cardiovascular prevention. Post-hoc analysis of the NID-2 trial. Cardiovasc Diabetol.

