# Supplementary material for: Glycemic control and cardiovascular outcomes in patients with diabetes and coronary artery disease according to triglyceride-glucose index: a large-scale cohort study
Source: Cardiovasc Diabetol. 2024 Jan 6;23:11. doi: 10.1186/s12933-023-02112-y (PMC10771684; doi:10.1186/s12933-023-02112-y)
Supplement: Supplementary file 1 — Supplementary Material 1: Table S1. Correlation analysis between TyG index and clinical risk factors. Table S2. Baseline characteristics according to CV events. Table S3. TyG tertiles in relation to secondary endpoints. Table S4. Glycemic control status in relation to study endpoints according to TyG index tertiles. Figure S1. Restrict cubic spine analysis for the association of TyG index with the risk of (A) CV events and (B) MACEs [file 12933_2023_2112_MOESM1_ESM.docx]

**Glycemic control and cardiovascular outcomes in patients with diabetes and coronary artery disease according to triglyceride-glucose index: a large-scale cohort study**

**Supplementary Material**

**Table S1** Correlation analysis between TyG index and clinical risk factors

| Variables | Correlation coefficient (r) | *P* value |
| --- | --- | --- |
| Age, years | -0.146 | <0.001 |
| BMI, kg/m^2^ | 0.142 | <0.001 |
| HbA1c, % | 0.322 | <0.001 |
| FBG, mmol/L | 0.592 | <0.001 |
| TC, mmol/L | 0.384 | <0.001 |
| TG, mmol/L | 0.778 | <0.001 |
| LDL-C, mmol/L | 0.304 | <0.001 |
| HDL-C, mmol/L | -0.233 | <0.001 |
| hsCRP, mg/L | 0.095 | <0.001 |
| Serum creatinine, μmol/L | 0.006 | <0.001 |

Abbreviations as in Table 1.

**Table S2** Baseline characteristics according to CV events

| **Characteristics ^a^** | **Overall**  **N = 9996** | **Non-event**  **N = 9615** | **Event**  **N = 381** | ***P* value** |
| --- | --- | --- | --- | --- |
| TyG index | 9.19±0.63 | 9.18±0.63 | 9.28±0.67 | 0.002 |
| Age, years | 60.29±9.27 | 60.19±9.23 | 62.93±9.82 | <0.001 |
| Male | 7502 (75.1) | 7218 (75.1) | 284 (74.5) | 0.862 |
| BMI | 26.31±3.21 | 26.32±3.20 | 26.07±3.52 | 0.141 |
| Clinical presentation |  |  |  | 0.007 |
| CCS | 3790 (37.9) | 3671 (38.2) | 119 (31.2) |  |
| ACS | 6206 (62.1) | 5944 (61.8) | 262 (68.8) |  |
| Family history of CAD | 1172 (11.7) | 1126 (11.7) | 46 (12.1) | 0.893 |
| Prior MI | 2623 (26.2) | 2477 (25.8) | 146 (38.3) | <0.001 |
| Prior revascularization ^b^ | 3047 (30.5) | 2908 (30.2) | 139 (36.5) | 0.011 |
| Hypertension | 6945 (69.5) | 6652 (69.2) | 293 (76.9) | 0.002 |
| Prior stroke | 1497 (15.0) | 1416 (14.7) | 81 (21.3) | 0.001 |
| PAD | 743 (7.4) | 701 (7.3) | 42 (11.0) | 0.009 |
| Current smoker | 3053 (30.5) | 2935 (30.5) | 118 (31.0) | 0.898 |
| CKD | 229 (2.3) | 202 (2.1) | 27 (7.1) | <0.001 |
| LVEF, % | 61.57±6.85 | 61.68±6.71 | 58.74±9.35 | <0.001 |
| **Laboratory tests** |  |  |  |  |
| Serum creatinine, μmol/L | 82.89±18.11 | 82.62±17.63 | 89.72±26.83 | <0.001 |
| eGFR, ml/min/m^2^ | 85.27±18.65 | 85.50±18.50 | 79.33±21.30 | <0.001 |
| HbA1c, % | 7.41±1.29 | 7.39±1.28 | 7.74±1.43 | <0.001 |
| FBG, mmol/L | 8.12±2.80 | 8.08±2.76 | 9.06±3.52 | <0.001 |
| TG, mmol/L | 1.83±1.24 | 1.83±1.24 | 1.83±1.28 | 0.945 |
| TC, mmol/L | 4.00±1.07 | 4.00±1.07 | 4.02±1.05 | 0.711 |
| HDL-C, mmol/L | 1.07±0.28 | 1.07±0.28 | 1.05±0.30 | 0.098 |
| LDL-C, mmol/L | 2.38±0.90 | 2.38±0.90 | 2.41±0.87 | 0.508 |
| hsCRP, mg/L | 2.66±3.03 | 2.65±3.02 | 3.01±3.25 | 0.003 |
| **Angiographic data** |  |  |  |  |
| SYNTAX score | 12.71±5.47 | 12.71±5.49 | 12.81±4.95 | 0.156 |
| Left main disease | 849 (8.5) | 813 (8.5) | 36 (9.4) | 0.556 |
| Three-vessel disease | 4765 (47.7) | 4565 (47.5) | 200 (52.5) | 0.061 |
| CTO lesion | 1056 (10.6) | 1014 (10.5) | 42 (11.0) | 0.832 |
| Thrombotic lesion | 202 (2.0) | 186 (1.9) | 16 (4.2) | 0.004 |
| Ostial lesion | 1205 (12.1) | 1154 (12.0) | 51 (13.4) | 0.463 |
| Type B2/C lesion | 7460 (74.6) | 7162 (74.5) | 298 (78.2) | 0.114 |
| Severe calcification | 359 (3.6) | 339 (3.5) | 20 (5.2) | 0.102 |
| **Medications** |  |  |  |  |
| Aspirin | 7351 (73.5) | 7080 (73.6) | 271 (71.1) | 0.304 |
| Statins | 9688 (96.9) | 9322 (97.0) | 366 (96.1) | 0.404 |
| ACEI/ARB | 2841 (28.4) | 2728 (28.4) | 113 (29.7) | 0.625 |
| β-blocker | 8981 (89.8) | 8635 (89.8) | 346 (90.8) | 0.582 |
| Diabetic therapy |  |  |  |  |
| Diet control | 892 (8.9) | 865 (9.0) | 27 (7.1) | 0.234 |
| Oral medication | 5081 (50.8) | 4893 (50.9) | 188 (49.3) | 0.590 |
| Insulin use | 1756 (17.6) | 1676 (17.4) | 80 (21.0) | 0.084 |

^a^ Values are expressed as mean ± standard deviation and count (percentage).

^b^ revascularization included percutaneous coronary intervention and coronary artery bypass grafting.

Abbreviations as in Table 1.

**Table S3** TyG tertiles in relation to secondary endpoints

| **TyG tertiles** | **Events (%)** | **Univariable analysis** | | **Multivariable analysis** | |
| --- | --- | --- | --- | --- | --- |
|  |  | **HR (95%CI)** | ***P* value** | **HR (95%CI)** | ***P* value** |
| CV death |  |  |  |  |  |
| T1 | 46 (1.4) | Reference | - | Reference | - |
| T2 | 56 (1.7) | 1.21 (0.82-1.79) | 0.334 | 1.41 (0.93-2.14) | 0.103 |
| T3 | 55 (1.6) | 1.19 (0.81-1.77) | 0.377 | 1.52 (0.90-2.59) | 0.120 |
| Nonfatal MI |  |  |  |  |  |
| T1 | 38 (1.1) | Reference | - | Reference | - |
| T2 | 36 (1.1) | 0.94 (0.60-1.49) | 0.800 | 0.87 (0.54-1.41) | 0.576 |
| T3 | 59 (1.8) | 1.55 (1.03-2.34) | 0.034 | 1.49 (0.87-2.56) | 0.144 |
| Nonfatal stroke |  |  |  |  |  |
| T1 | 18 (0.5) | Reference | - | Reference | - |
| T2 | 18 (0.5) | 1.00 (0.52-1.91) | 0.990 | 1.00 (0.51-1.98) | 0.998 |
| T3 | 18 (0.5) | 1.00 (0.52-1.92) | 0.995 | 0.90 (0.40-2.07) | 0.811 |

Abbreviations as in Table 1 and Table 2.

**Table S4 Glycemic control status in relation to study endpoints according to TyG index tertiles**

| **TyG tertiles** | **Glycemic control**  **Events (%)** | | **Univariable analysis** | | **Multivariable analysis ^a^** | |
| --- | --- | --- | --- | --- | --- | --- |
|  | **Controlled** | **Uncontrolled** | **HR (95%CI)** | ***P* value** | **HR (95%CI)** | ***P* value** |
| **CV death** | 57 (1.3) | 100 (1.8) | 0.72 (0.52-1.00) | 0.047 | 0.72 (0.51-1.01) | 0.057 |
| TyG T1 | 25 (1.3) | 21 (1.5) | 0.83 (0.46-1.48) | 0.520 | 0.79 (0.43-1.48) | 0.468 |
| TyG T2 | 23 (1.5) | 33 (1.8) | 0.85 (0.50-1.45) | 0.557 | 1.00 (0.55-1.79) | 0.988 |
| TyG T3 | 9 (0.9) | 46 (1.9) | 0.49 (0.24-0.99) | 0.049 | 0.59 (0.28-1.23) | 0.157 |
| **Nonfatal MI** | 40 (0.9) | 93 (1.7) | 0.54 (0.37-0.78) | 0.001 | 0.59 (0.41-0.87) | 0.007 |
| TyG T1 | 16 (0.8) | 22 (1.6) | 0.50 (0.26-0.96) | 0.036 | 0.55 (0.28-1.08) | 0.082 |
| TyG T2 | 14 (0.9) | 22 (1.2) | 0.78 (0.40-1.52) | 0.464 | 0.91 (0.44-1.89) | 0.808 |
| TyG T3 | 10 (1.1) | 49 (2.1) | 0.51 (0.26-1.00) | 0.050 | 0.53 (0.27-1.07) | 0.075 |
| **Nonfatal stroke** | 22 (0.5) | 32 (0.6) | 0.87 (0.50-1.49) | 0.608 | 0.85 (0.49-1.50) | 0.583 |
| TyG T1 | 12 (0.6) | 6 (0.4) | 1.39 (0.52-3.70) | 0.510 | 1.37 (0.49-3.87) | 0.548 |
| TyG T2 | 5 (0.3) | 13 (0.7) | 0.47 (0.17-1.31) | 0.150 | 0.44 (0.14-1.34) | 0.147 |
| TyG T3 | 5 (0.5) | 13 (0.5) | 0.96 (0.34-2.69) | 0.938 | 0.82 (0.29-2.36) | 0.716 |

^a^ Models adjusted for age, male sex, BMI, hypertension, diabetes mellitus, ACS, previous MI, previous revascularization, current smoker, previous stroke, PAD, LVEF, TC, LDL-C, eGFR, hsCRP, SYNTAX score, left main disease, three-vessel disease, type B2/C lesion, CTO lesion, aspirin use, and statins use.

Abbreviations as in Table 1 and Table 2.

**Figure S1** Restrict cubic spine analysis ^a^ for the association of TyG index with the risk of (A) CV events and (B) MACEs.


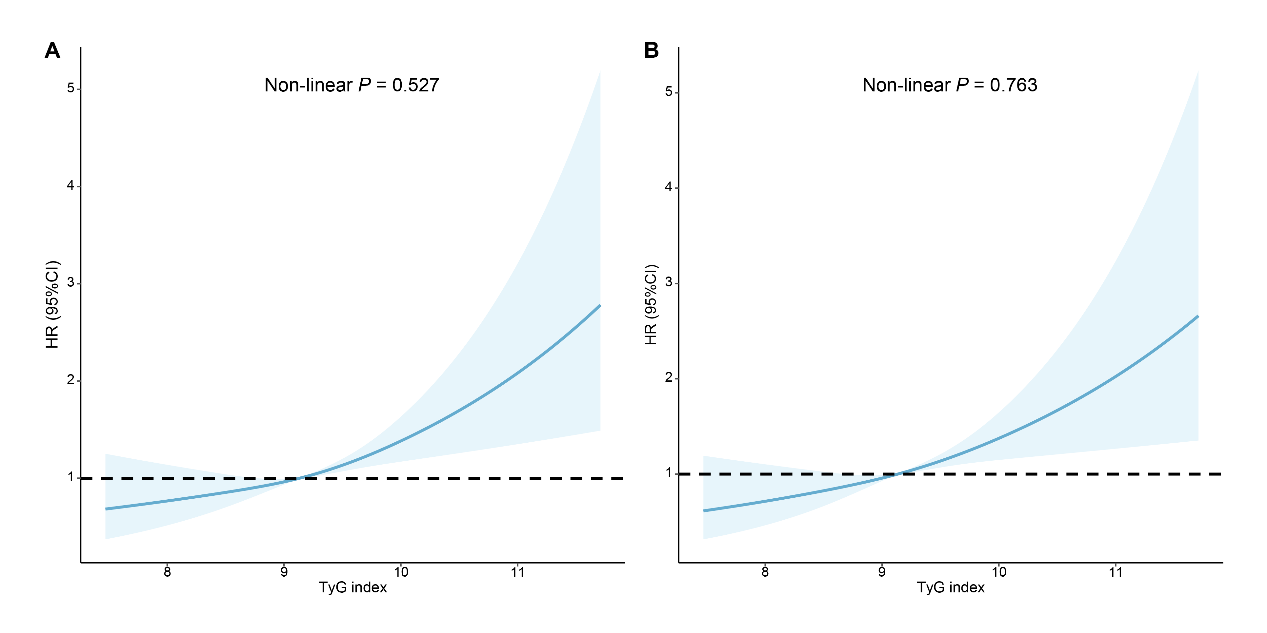


^a^ Models adjusted for age, and male sex.

Abbreviations as in Table 1 and Table 2.
